# Hypothermia and pediatric cardiac arrest

**DOI:** 10.4103/0974-2700.66533

**Published:** 2010

**Authors:** Michelle L Schlunt, Lynn Wang

**Affiliations:** Department of Anesthesiology, Loma Linda University School of Medicine, California, USA

**Keywords:** Cardiac arrest, hypothermia, neuroprotection, pediatric

## Abstract

The survival outcome following pediatric cardiac arrest still remains poor. Survival to hospital discharge ranges anywhere from 0 to 38% when considering both out-of-hospital and in-hospital arrests, with up to 50% of the survivors having neurologic injury. The use of mild induced hypothermia has not been definitively proven to improve outcomes following pediatric cardiac arrest. This may be due to the lack of consensus regarding target temperature, best method of cooling, optimal duration of cooling and identifying the patient population who will receive the greatest benefit. We review the current applications of induced hypothermia in pediatric patients following cardiac arrest after searching the current literature through Pubmed and Ovid journal databases. We put forth compiled recommendations/guidelines for initiating hypothermia therapy, its maintenance, associated monitoring and suggested adjunctive therapies to produce favorable neurologic and survival outcomes.

## INTRODUCTION

The use of induced therapeutic hypothermia for neuroprotection against hypoxic ischemic brain injury has been researched over the last 30–40 years. It is currently recommended for the post-resuscitation treatment of ventricular fibrillation cardiac arrest based on two landmark clinical trials in 2002 that confirmed improved survival and neurologic outcome in adults.[1,2] For the pediatric population, the use of therapeutic hypothermia is infrequent due to prior reports of lack of outcome improvement and increased infectious complications when used in near-drowning victims.[3,4] There has been a renewed interest in its use, however, due to the findings in the adult population and improved survival[5] and neurologic outcome in newborns following perinatal asphyxia.[6,7] Here, we review the current literature searched through Pubmed and Ovid journal databases.

## EPIDEMIOLOGY OF PEDIATRIC CARDIAC ARREST

The pediatric guidelines, published in October 1995, define cardiac arrest as “the cessation of cardiac mechanical activity, determined by the inability to palpate a central pulse, unresponsiveness and apnea.”[8] Cardiopulmonary arrest is however rare in children. The overall population-based incidence of non-traumatic pediatric out-of-hospital cardiac arrest is 8 per 100,000 pediatric person-years compared to 126 per 100,000 adult patient-years.[9] When broken down by age and gender, infants <1 year of age and males have the highest incidence.[9–11] Pediatric cardiac arrest more commonly results from respiratory failure or circulatory shock as opposed to arrhythmia in adults. This is why many practitioners find it difficult to simply extrapolate the results with hypothermia in the adult population to the pediatric population. However, 7% of the children experiencing an out-of-hospital cardiac arrest had an initial cardiac rhythm of ventricular tachycardia/ventricular fibrillation (VT/VF).[9] The incidence of VT/VF increases with increasing age.[9,12] Survival to hospital discharge with an initial rhythm of VT/VF is higher compared to those children with an initial rhythm of asystole or pulseless electrical activity (PEA).[9] Children greater than 1 year of age are more likely to survive to hospital discharge compared to infants and adults.[9] The latest survival rates to hospital discharge for those children who received emergency medical service treatment are: infants <1 year of age 3.5%, children 1–11 years of age 10.4% and adolescents 12–19 years of age 12.6%.[9] Neurological outcomes remain poor, with death being attributed to neurologic futility or brain death in 69% of out-of-hospital cardiac arrests.[13]

In-hospital cardiac arrests of children admitted to a pediatric intensive care unit occur at a rate of 0.94 cardiac arrests per 100 admissions.[14] Pediatric patients suffering an in-hospital cardiac arrest differ from the out-of-hospital cardiac arrest subpopulation due to a chronic pre-existing condition being present twice as often and a cardiac etiology more likely as the cause of the arrest.[13] Initial VT/VF occurs in 10% of the in-hospital cardiac arrests.[12] As in out-of-hospital pediatric cardiac arrest, initial VT/VF is associated with a higher rate of survival to hospital discharge compared to those without VT/VF.[12] Extracorporeal membrane oxygenation (ECMO) initiated within 24 h after cardiac arrest is associated with a decrease in hospital mortality.[14] There is a lower incidence of mortality and greater likelihood of good neurologic outcome with an in-hospital cardiac arrest versus out-of-hospital cardiac arrest.[13] This is probably related to the fact that out-of-hospital pediatric cardiac arrests are usually not witnessed events and so the length of time to initiation of resuscitation is prolonged, and that only about one-third of the arrest victims receive by-stander cardiopulmonary resuscitation (CPR)[15] as many spectators are afraid of performing CPR wrongly. Survival also depends on other factors such as actual duration of CPR, quality of CPR administered and the extent of necessary pharmacologic intervention needed during CPR.

## MECHANISMS OF ACTION FOR INDUCED HYPOTHERMIA

Is there a role for induced hypothermia in the post-resuscitation phase of pediatric cardiac arrest? Hypothermia has been a long-established neuroprotective strategy in cardiopulmonary bypass for both adult and pediatric patients. The 2006 International Liaison Committee on Resuscitation treatment recommendations states, “induction of hypothermia (32-34°C) for 12 to 24 hours should be considered in children who remain comatose after resuscitation from cardiac arrest.”[16] What are the potential beneficial effects with induced hypothermia that could be applied to the post-resuscitation cardiac arrest pediatric population?

With the onset of cardiac arrest, cerebral perfusion ceases. In many cases, pediatric cardiac arrest is related to a respiratory etiology such as drowning or choking[17] and therefore a period of hypoxia precedes the cessation of cerebral blood flow compounding the neurologic insult. Unfortunately, the spontaneous return of circulation does not occur in up to 70% of out-of-hospital cardiac arrests and in up to 50% of in-hospital cardiac arrests.[18] If spontaneous return of circulation does occur, a period of cerebral hyperperfusion occurs that can lead to inflammation, mitochondrial dysfunction, calcium dysregulation, increased oxidative stress, metabolic failure, initiation of excitotoxic pathways and cellular apoptosis. This period is then followed by delayed hypoperfusion. Many children post-resuscitation remain hemodynamically unstable due to myocardial stunning and vasodilatation requiring inotropic and vasopressor agents for cardiovascular support. This hemodynamic instability can cause further secondary neurologic injury. Because of the continuum of brain development in the pediatric population, there are age-related responses to cerebral hypoxia/injury,[19] such as selective neuronal vulnerability.[20] The utilization of electroencephalographic (EEG) monitoring may be beneficial during this period to assess and predict neurologic activity in the comatose child.[21]

There are a number of molecular mechanisms with which hypothermia can influence neuronal injury. These have been mainly demonstrated in animal models. Mild hypothermia has been shown to protect against neuronal loss in gerbils.[22] Hypothermia is well known for its effect on decreasing the cerebral metabolic rate by 6% for each 1°C drop in temperature, delaying the depletion of energy stores and the subsequent change from aerobic to anaerobic energy metabolism. With the fall in energy stores, the release of excitatory neurotransmitters such as N-methyl-D-aspartate (NMDA)[23] occurs and mitochondrial dysfunction also develops. The consequences of mitochondrial dysfunction are numerous and include increased oxidative stress and excessive intracellular calcium leading to the activation of a host of proteases and protein kinases responsible for eventual neuronal cell death.[24] Hypothermia has been shown to attenuate a number of these processes,[25] reduce free radical formation[26] and decrease oxidative stress. With the inflammatory response associated with reperfusion leading to neuronal swelling, hypothermia decreases leukocyte endothelial interactions and has anti-inflammatory effects.[27]

## RECOMMENDATIONS

There are a number of questions regarding hypothermia therapy due to lack of consensus in the pediatric medical community mainly because there are no randomized-controlled studies available. What is the optimal way to cool and to what temperature? How long should we really maintain hypothermia? What routine monitoring should be done during hypothermia therapy? Do we need to actively re-warm, and if so how fast? These are just a few of the controversial issues. Currently, there are no standardized measures for inducing hypothermia in the pediatric population. What one institution has adopted for themselves is not necessarily what is being followed at other institutions. We have attempted to compile recommendations/guidelines based on the small number of studies and case reports utilizing hypothermia in pediatric patients after cardiac arrest.

It is recommended to initiate cooling as quickly as possible as it is undetermined how long after pediatric cardiac arrest cooling can be applied and still be effective. It is suggested that hypothermia be achieved within 2–6 h of the insult to be beneficial.[28,29] Some authors suggest initiating cooling measures prior to hospital arrival if possible for an out-of-hospital cardiac arrest. However, this may prove difficult to accomplish due to issues of consent.[30] Cooling methods include cooling mattresses, forced air-cooling blankets and ice packs for a target temperature of 32–34°C. The use of ice-cold normal saline gastric lavage in adolescents[20] and intravenous ice-cold normal saline in adults[31,32] has been described. If available at the treatment center, ECMO can be used as a cooling strategy,[33] which has been associated with an increase in survival to hospital discharge in children with prolonged cardiopulmonary arrest duration.[34] The subpopulation that appears to benefit most thus far from ECMO is children with pre-existing cardiac disease who suffered an in-hospital cardiac arrest.[33] It is unknown whether whole-body hypothermia is necessary or just selective head cooling as used in neonates.[35] What is the optimal site to monitor for temperature: rectal, bladder, esophageal or tympanic membrane to avoid over-cooling?[36] During deep hypothermic circulatory arrest, the esophageal temperature is considered an approximation of brain temperature while the rectal temperature in considered to be the core temperature. The use of bladder temperature as core temperature requires adequate urine output. The use of muscle relaxants to avoid shivering, which increases myocardial oxygen consumption, has the drawback of interfering with neurologic assessment. The use of EEG monitoring during hypothermia in children has been utilized in this situation. Forty-seven percent of the patients experienced seizures noted on EEG and 32% had EEG evidence of non-convulsive status epilepticus.[37] Children with severely abnormal EEG backgrounds had seizures more frequently and had poorer neurologic outcomes.[37] The majority of seizures were generalized and it was suggested that a limited montage, such as the BIS Pediatric^®^, might be easy to use in institutions where a bedside EEG technologist is not available.[37]

The optimal duration of induced hypothermia is unknown. Currently, most treatment centers that report utilizing induced hypothermia recommend 12–24 h, and infrequently up to 48 h.[38] A longer duration of hypothermia may be needed if there was a delay in initiating cooling.[39] EEG monitoring during hypothermia therapy and the measurement of biochemical markers such as neuron-specific enolase and S-100B[40] may help to serve as prognostic indicators to provide guidance for prolonging hypothermia treatment. Neuron-specific enolase levels elevated at 48 h after arrest are associated with poor neurologic outcome while elevated S-110B levels are associated with mortality.[40] Neuroimaging studies such as magnetic resonance imaging or computed tomography in conjunction with serial neurologic examinations also aid the guidance of continued medical therapy. Medical care providers are encouraged to use standard measures of assessment of neurologic injury following pediatric cardiac arrest, such as the Pediatric Cerebral Performance Category scoring system for estimating neurocognitive status, which employs a six-point system: 1 = normal, 2 = mild disability, 3 = moderate disability, 4 = severe disability, 5 = coma or vegetative state, 6 = death.

The issue of rewarming is also controversial. Children post-resuscitation may experience spontaneous hypothermia due to disturbances in temperature autoregulation.[41,42] These children when actively rewarmed are seen to exceed target-warming temperature and become hyperthermic, which can exacerbate neurologic injury.[41] If rewarming is chosen, suggestive recommendations include warming no faster than 0.5–1°C/h.[38] Existing hypotension may be exacerbated due to the associated vasodilatation with rewarming. Ninety-one percent of the respondents to a survey on the use of hypothermia in comatose children following cardiac arrest reported that they did not actively rewarm.[38]

Despite 65% of pediatric care providers being aware of the benefits of hypothermia post-resuscitation in adults after cardiac arrest, only 9% reported using hypothermia on a consistent basis in comatose children following cardiac arrest.[38] However, the vast majority also reported their willingness to participate in a study randomizing pediatric cardiac arrest victims to hypothermia or normothermia, and to the use of standardized post-resuscitation treatment protocols.[38] The use of a goal-directed standardized protocol addressing hemodynamic management, ventilation and glucose management has already been shown to improve survival.[43]

Induced hypothermia is surely not the only treatment approach to improving survival and neurologic outcome following pediatric cardiac arrest. To increase the chances of a favourable neurologic outcome, induced hypothermia should probably be combined with other adjunctive therapies that together could act synergistically. Pharmacologic approaches include steroids, NMDA antagonists, antiinflammatory agents or antiexcitotoxic agents.[44,45] Seizure prophylaxis with anticonvulsants could be considered as well due to the high incidence of seizure activity seen with EEG monitoring.[37] These pharmacologic therapies would be easier to initiate out in the field following out-of-hospital cardiac arrests compared to hypothermia.

Complications related to hypothermia treatment include ventricular arrhythmias, infection,[3,4] coagulation and hemodynamic instability due to myocardial depression. Many of these complications are associated with greater depths of hypothermia such as deep hypothermic circulatory arrest with temperatures down to 17°C as opposed to the mild hypothermia target of 32–34°C. Many of the studies thus far have not demonstrated an increased incidence of these complications in their treatment groups receiving hypothermia.[46–48]

Prevention of cardiac arrest is really a key fundamental. Not all cardiac arrests can obviously be prevented, but other incorporated strategies such as the implementation of medical emergency treatment teams in hospital institutions have been shown to decrease the incidence of preventable cardiac arrests and improve survival.[49] There are many areas related to pediatric cardiac arrest, which, if improved, most certainly will aid in improving survival outcomes. Areas such as the continued education of medical providers stressing the importance of providing effective conventional CPR (“push hard, push fast, minimize interruptions; allow full chest recoil, and don’t hyperventilate”)[50] along with early rhythm recognition of VT/VF and the prompt use of defibrillation[51] as mortality increases by 7–10% per minute of delay to defibrillation.[52] These principles and skills can be learned through the use of simulation training.[52] The use of frequent repeat in-training sessions can prevent these learned skills from being forgotten.[52] We should encourage medical providers to always think ahead and possibly organize the rapid deployment of ECMO within 30 min of arrest[53] when available and applicable, taking the initiative to formulate and incorporate goal-directed post-resuscitation protocols addressing hemodynamics, ventilation, glucose and temperature management and finding innovative and creative ways to promote community education to improve the participation of by-stander CPR for out-of-hospital arrests. The answers to many issues related to improving survival and neurologic outcome after pediatric cardiac arrest continue to remain obscure, but hopefully with the use of compiling databases and the planned Therapeutic Hypothermia After Pediatric Cardiac Arrest (THAPCA) clinical trial, we may find some truly definitive answers.

## CONCLUSIONS

Overall, survival to hospital discharge after pediatric cardiac arrest now has surpassed adult survival (6.4% versus 4.5%).[9] It is not, however, simply survival rates that need to be improved. It is devastating to have a child survive a cardiac arrest only to remain in a persistent vegetative state. We also need to focus investigation into therapeutic approaches to improve neurologic outcomes. The use of induced hypothermia may turn out to be one such therapeutic approach. The evidence still remains sparse and limited in nature. With the planned THAPCA clinical trial, hopefully, we can gain a better understanding of the influence of induced hypothermia on the developing brain after cardiac arrest.
